# Straightforward and sensitive RT-qPCR based gene expression analysis of FFPE samples

**DOI:** 10.1038/srep21418

**Published:** 2016-02-22

**Authors:** Fjoralba Zeka, Katrien Vanderheyden, Els De Smet, Claude A. Cuvelier, Pieter Mestdagh, Jo Vandesompele

**Affiliations:** 1 Center for Medical Genetics, Ghent University, Belgium; 2 Cancer Research Institute Ghent, Ghent University, Belgium; 3 Department of Pathological Anatomy, Ghent University Hospital, Belgium

## Abstract

Fragmented RNA from formalin-fixed paraffin-embedded (FFPE) tissue is a known obstacle to gene expression analysis. In this study, the impact of RNA integrity, gene-specific reverse transcription and targeted cDNA preamplification was quantified in terms of reverse transcription polymerase chain reaction (RT-qPCR) sensitivity by measuring 48 protein coding genes on eight duplicate cultured cancer cell pellet FFPE samples and twenty cancer tissue FFPE samples. More intact RNA modestly increased gene detection sensitivity by 1.6 fold (earlier detection by 0.7 PCR cycles, 95% CI = 0.593–0.850). Application of gene-specific priming instead of whole transcriptome priming during reverse transcription further improved RT-qPCR sensitivity by a considerable 4.0 fold increase (earlier detection by 2.0 PCR cycles, 95% CI = 1.73–2.32). Targeted cDNA preamplification resulted in the strongest increase of RT-qPCR sensitivity and enabled earlier detection by an average of 172.4 fold (7.43 PCR cycles, 95% CI = 6.83–7.05). We conclude that gene-specific reverse transcription and targeted cDNA preamplification are adequate methods for accurate and sensitive RT-qPCR based gene expression analysis of FFPE material. The presented methods do not involve expensive or complex procedures and can be easily implemented in any routine RT-qPCR practice.

RNA expression analysis by reverse transcription quantitative polymerase chain reaction (RT-qPCR) has been a key contributing technology in current genomic and molecular biomarker research^1^,^2^. qPCR-based technologies have quickly evolved from single–gene to large-scale screenings, greatly contributing to characterization and categorization of disease^3^,^4^. Large-scale screening studies also require many patients, creating an increased demand for human tissue samples^5^,^6^. While fresh frozen tissue samples are the preferred starting material for RT-qPCR, the availability of such biomaterial is generally limited^7^. Current clinical practice is not well equipped for standardized processing and storage of fresh-frozen patient material. Often, only small tissue volumes are procured, or the biomaterial is entirely used for routine preparation of formalin-fixed and paraffin-embedded (FFPE) material, intended for downstream histological examination^8^,^9^.

One of the benefits of FFPE samples is that these are stable at room temperature and amenable for long-term storage^10^. Standardized methods for FFPE preparation have led to accumulation of large FFPE sample archives worldwide. In addition, availability of long-term clinical follow-up data makes these archives particularly attractive. Nevertheless, gene expression analysis on FFPE samples is challenging because RNA extracted from FFPE material is of very poor quality, impairing detection sensitivity^7^,^11^,^12^.

During formalin fixation, RNA is cross-linked to adjacent molecules (DNA and proteins) by addition of methylol groups and methylene bridge formation^13^. Crosslink dissociation during RNA purification leads to severe fragmentation^14^. RNA fragmentation and residual chemical modifications on the RNA molecules hamper primer annealing and elongation during reverse transcription and PCR amplification, resulting in low qPCR sensitivity and reduced reproducibility.

Several studies have proposed ways to increase the success of qPCR experiments in FFPE material. For instance, heat application and proteinase K digestion on FFPE sections results in recovery of less fragmented RNA with fewer chemical modifications^13^,^15^,^16^. Reverse transcription with random hexamers instead of oligo-dT primers results in higher cDNA conversion coverage^17^. qPCR primers for generation of short amplicons were proven to increase gene detection rate in fragmented RNA^18^,^19^,^20^. Limited-cycle preamplification has been used to compensate for decreased sensitivity by increasing the amount of specific gene targets prior to qPCR^21^,^22^. Despite these individual improvements, the application of RT-qPCR on FFPE material remains challenging and not often applied. Further, there is no consensus strategy for straightforward and reliable RNA expression measurement by RT-qPCR in FFPE samples.

In this study, we tested and quantified to what extent RNA integrity, reverse transcription and preamplification impact qPCR sensitivity when using FFPE material. Our results give researchers the possibility to evaluate each of the suggested RT-qPCR steps and to choose for the best design adapted to their study. The applied methods do not involve expensive or complex technologies and can easily be implemented in any laboratory equipped for routine RT-qPCR measurements.

## Results

### RNA isolation method affects RNA yield, quality and RT-qPCR sensitivity

**Figure 1:**
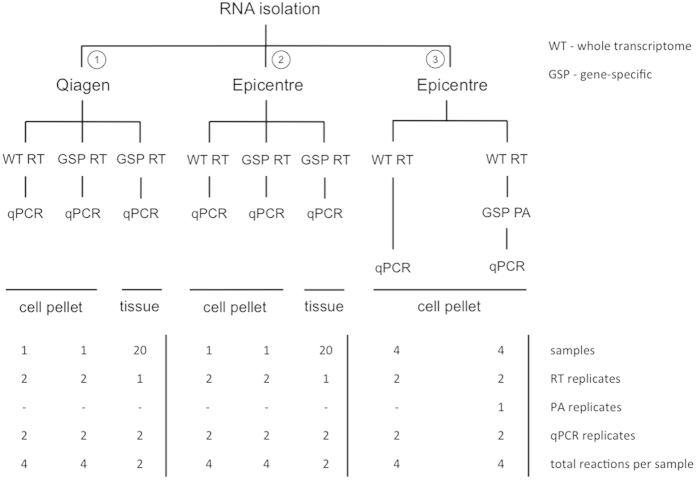
Schematic representation of the three RT-qPCR workflows evaluated in this study, with indication of number of samples and replicates. WT RT = whole transcriptome reverse transcription; GSP RT = gene-specific reverse transcription; GSP PA = gene-specific preamplification. Two RNA isolation kits were tested (Qiagen and Epicentre). In workflow 1 (Qiagen) and 2 (Epicentre) whole transcriptome reverse transcriptions was evaluated against gene-specific reverse transcription. In workflow 3 the performance of gene-specific preamplification was evaluated.

**Figure 2:**
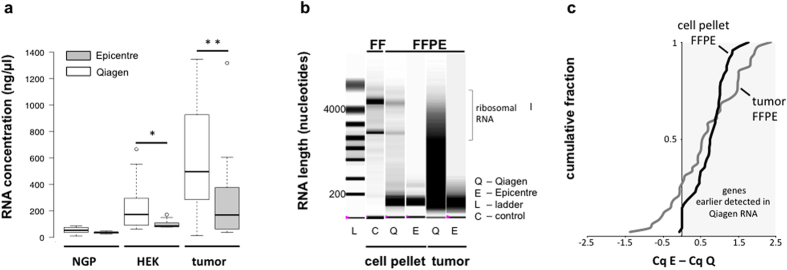
Effect of RNA purification method on RNA concentration and integrity. (**a**) RNA concentration of FFPE samples processed with Epicentre (E) or Qiagen (Q). Each boxplot represents 6 data points for NGP and HEK-293T FFPE cell pellets, and 20 data points for tumor FFPE samples. Statistical significance between Qiagen and Epicentre samples is calculated by 2-tailed paired t-test: (*) < 0.05, (**) < 0.01. Whiskers extend to data points that are less than 1.5× the inter quartile range away from 1st/3rd quartile. Horizontal line depicts the median-value. (**b**) Representative examples of microfluidic electrophoresis lanes for FF (fresh frozen) and FFPE (formalin-fixed paraffin-embedded) samples (L = ladder and C = high-quality RNA control). In this picture HEK-293T FFPE sample is shown. (**c**) Cumulative fraction of qPCR cycle difference (Cq from Epicentre samples minus Cq from Qiagen samples) for cell pellet FFPE (HEK-293T cell line) and tumor FFPE. Positive ∆Cq values point at higher expression (earlier detection, lower Cq) in Qiagen extracted RNA samples.

Two RNA isolation methods (Qiagen, Epicentre) were used to isolate RNA from FFPE sections. Three parallel rounds of RNA extraction were performed on equal numbers of FFPE sections (5 or 8 sections, see Materials and Methods and Fig. 1). Significantly higher RNA concentration and yield were observed for the 6 cell pellet FFPE samples (P < 0.05) and 20 FFPE clinical tumor samples (P < 0.01) extracted with the Qiagen kit (Fig. 2a, Supplemental Table 1 and 2). On average, the RNA concentration was 3.25 fold higher (95% CI = 2.19–4.30) for FFPE samples processed with the Qiagen kit. Samples isolated with the Epicentre protocol resulted in shorter RNA fragments, both for cell line FFPE and tumor FFPE samples (Fig. 2b, Supplemental Fig. 1). We performed RT-qPCR to measure expression of 48 universally expressed genes on both RNA isolates. The average Cq value was 0.686 cycles lower in Qiagen RNA (95% CI = 0.593–0.850, P < 0.001), which corresponds to 1.61 fold increase in sensitivity. Earlier detection was observed for 73% and 85% of the tested genes in tumor and cell line FFPE samples, respectively (Fig. 2c).

### Multiplex gene-specific RT improves sensitivity of RT-qPCR in FFPE

**Figure 3:**
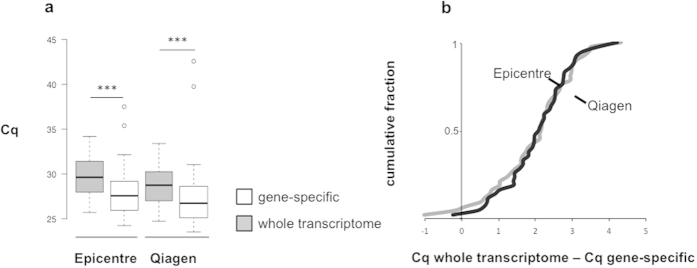
Effect of gene-specific RT and whole transcriptome RT on RT-qPCR sensitivity. (**a**) Cq values for HEK-293T cell pellet FFPE samples processed with gene-specific RT or whole transcriptome RT. Each boxplot represents average Cq values from two RNA samples for 48 genes, shown for Epicentre RNA and Qiagen RNA isolates. Differences between gene-specific RT and whole transcriptome RT are significant (2-tailed paired t-test), (***) < 0.001. Whiskers extend to data points that are less than 1.5× the inter quartile range away from 1st/3rd quartile. Horizontal line depicts median value. (**b**) Cumulative fraction of qPCR cycle difference (Cq from whole transcriptome RT minus gene-specific RT Cq). Positive ∆Cq values point at genes that are earlier detected (lower Cq) by gene-specific RT.

Since oligo-dT mediated priming of FPPE RNA does not result in uniform cDNA conversion (due to its fragmented status and dislocation of poly-A tail from the rest of the transcript fragments) we decided to evaluate a 48-plex gene-specific cDNA synthesis. qPCR primers complementary to the target RNA sequence were used for reverse transcription reaction at 100 nM concentration. This approach was compared to whole transcriptome reverse transcription, where cDNA synthesis is prepared by a combination of oligo-dT and random primers (Fig. 3a). Gene-specific reverse transcription significantly increased qPCR sensitivity by a factor of 4.96 and 4.00 in Epicentre and in Qiagen samples, respectively, which corresponds to an average Cq decrease of 2.31 (95% CI = 1.99–2.63) and 2.03 cycles (95% CI = 1.73–2.32), respectively (P < 0.001). The vast majority of the genes (>95%) showed lower Cq values and more than 80% of the genes was detected at least one cycle earlier with gene-specific RT compared to whole transcriptome RT (Fig. 3b).

### Multiplex gene-specific pre-amplification maintains gene expression ratios in FF and FFPE samples and improves sensitivity of gene detection

**Figure 4:**
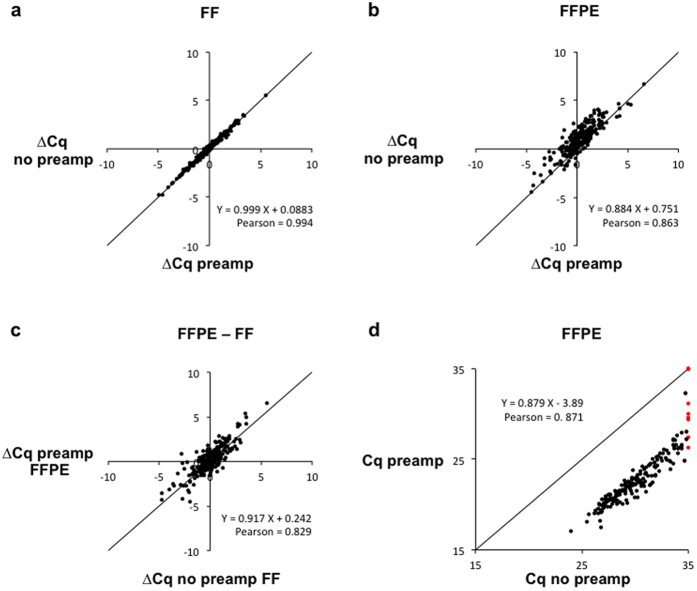
Effect of preamplification on qPCR quantification cycle difference (∆Cq) and qPCR sensitivity. ∆Cq is calculated as difference in Cq values between any two cell lines from four tested cell lines and 48 genes (totaling 6 × 48 data points). (**a**) ∆Cq Pearson correlation between preamplified and non-preamplified fresh frozen (FF) samples. (**b**) ∆Cq Pearson correlation between preamplified and non-preamplified cell pellet FFPE samples. (**c**) ∆Cq Pearson correlation between preamplified cell pellet FFPE samples and non-preamplified FF cell line samples. (**d**) Cq Pearson correlation between preamplified and non-preamplified samples from cell pellet FFPE samples. Cq values are shown for 4 cell lines and 48 genes, hence 4 × 48 data points were used in the correlation plot. Dots marked in red represent genes detected in preamplified samples only. No qPCR signal is observed for these genes in non-preamplified samples. For visualization purposes, undetected signal was replaced by a Cq value of 35.

We tested 48-plex gene-specific preamplification on whole transcriptome RT product (generated by combined oligo-dT and random priming). High-quality RNA from fresh frozen (FF) human cell line pellets and low-quality RNA from FFPE cell pellets was used. Only FFPE Epicentre isolates were used for this experiment, since these were most representative for archived tissue FFPE (higher degree of RNA fragmentation). The forward and the reverse primer from each of the 48 genes were pooled to obtain a 96-plex primer pool at 50 nM final concentration. For each gene, expression differences (∆Cq) were calculated between any two cell lines (from 4 tested cell lines) under 4 conditions, i.e. FF samples with and without preamplification and FFPE samples with and without preamplification. Conservation of differential expression was then evaluated by calculating Pearson’s correlation coefficients between the expression differences for preamplified and non-preamplified material, evaluated in FF samples and FFPE samples separately (Table 1). We observed significant positive correlations in both FF and FFPE samples when comparing workflows with and without preamplification (r = 0.994, P < 0.001 and r = 0.863, P < 0.001, respectively, Fig. 4a,b). These results suggest that differential gene expression analysis is not affected by introduction of preamplification in high quality RNA as well as in fragmented RNA samples. Comparison of expression ratios between FFPE and FF samples also resulted in high Pearson’s correlation (r = 0.829, P < 0.001) (Fig. 4c), showing that gene expression differences between samples are generally well maintained in FFPE material after the fixation procedure.

After having confirmed conservation of expression differences among preamplified or non-preamplified samples, the effect on qPCR sensitivity was evaluated. Using our workflow, the introduction of preamplification substantially improved sensitivity of gene detection by 172 fold in FFPE samples (average Cq decrease = 7.43, 95% CI = 6.83–7.05, P < 0.001) and by 102 fold in fresh frozen samples (average Cq decrease = 6.67, 95% CI = 6.56–6.72, P < 0.001). Without preamplification, 5% of the reactions were negative for a 35-cycle cutoff (Fig. 4d). If the detection cutoff was adjusted to 32 cycles, then 26% of the reactions were negative. With preamplification, these values were reduced to 3% (35-cycle cutoff) and 4% (32-cycle cutoff).

In order to evaluate the possible impact of the multiplex preamplification reaction on formation of non-specific by-product, we performed consecutive reverse transcription and preamplification on negative control MS2 phage RNA. We observed a positive qPCR signal below 35 cycles (non-specific qPCR product) for 19 out of 96 reactions (2 × 48 genes) with an average Cq-value of 31.9 (95% CI = 31.5–32.4). The non-specific signal is observed in the high Cq ranges with an average Cq difference of 15 and 10 cycles between the specific and non-specific target amplification, in FF and FFPE material, respectively.

**Figure 5:**
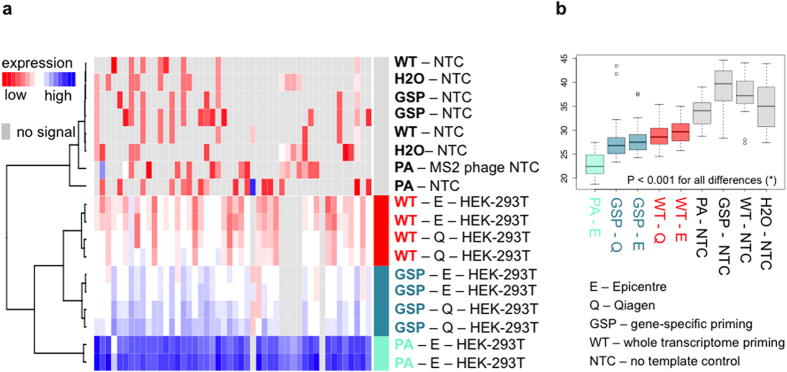
Overview of non-normalized gene expression (Cq values) in preamplified and non-preamplified samples processed with gene-specific RT and whole transcriptome RT. (**a**) Unsupervised hierarchical clustering of Cq values for 48 genes in 10 HEK-293T FFPE cell pellets and 8 no template controls (NTCs). Distance measure = Manhattan, clustering method = Ward. (**b**) Boxplot analysis for comparison of gene expression levels in preamplified samples (PA), non-preamplified gene-specific RT samples (GSP), non-preamplified whole transcriptome RT samples (WT) and no template controls (NTC), shown for Qiagen (Q) and Epicentre (E) RNA isolates. (*) All differences are significant (two-tailed paired t-test based, P < 0.001), with exception of differences within the NTC boxplot group. Whiskers extend to data points that are less than 1.5× the inter quartile range away from 1st/3rd quartile. Horizontal line depicts median value.

In ‘no template control’ (NTC) water samples for the qPCR, not preceded by reverse transcription and preamplification, only 3 out of 96 reactions showed non-specific signal (average Cq = 33.9, CI = 33.7–34.1, Fig. 5a).

### Comparison of RT-qPCR shensitivity

In order to summarize to what extent each of the above-described adjustments impacts RT-qPCR sensitivity, unsupervised hierarchical clustering and heat map analysis was performed on 10 HEK-293T samples based on Cq-values for the 48 measured genes (Fig. 5a): 2 samples underwent preamplification, 4 were processed with combined oligo-dT and random priming (whole transcriptome) and 4 underwent gene-specific RT (Table 2). Both Epicentre and Qiagen RNA isolates were included in gene-specific and whole-genome priming tests, whereas for the pre-amplification tests only Epicentre isolates were used. Eight no template control (NTC) reactions were included in the analysis: 2 NTC reactions for whole transcriptome reverse transcription, 2 for gene-specific reverse transcription, 2 for preamplification and 2 qPCR NTC reactions. In order to quantify to what extent the resulting sample clusters differ, paired student t-tests (2-tailed) were performed (Fig. 4b).

Four main clusters were observed; the first containing preamplified samples, the second non-preamplified samples treated by gene-specific RT, the third non-preamplified samples treated by whole transcriptome RT, and the fourth containing all negative controls. The average Cq difference is 4.34 (95% CI = 3.84–4,83, P < 0.001) between the preamplification cluster and the gene-specific RT cluster and 6.40 (95% CI = 5.95–6.81, P < 0.001) between the preamplification cluster and whole transcriptome RT cluster (Fig. 4b). This translates into 20.2 and 84.3 fold increased sensitivity when using preamplification compared to gene-specific RT and whole transcriptome RT, respectively.

If only the cluster with non-preamplified samples is considered, a larger distance is observed between samples processed with different RT priming strategy than samples processed with a different RNA isolation method (Fig. 4a). In fact, a significant difference in sensitivity of 4.03 fold is observed between samples processed with gene-specific RT and whole transcriptome RT (average ∆Cq = 2.01, 95% CI = 1.76–2.26, P < 0.001). This difference is larger than the difference between samples processed with different RNA isolation methods, resulting in at least 1.68 fold sensitivity difference (∆Cq = 0.75, 95% CI = 0.688–0.814 for gene-specific RT settings and, ∆Cq = 0.83, 95% CI = 0.715–0.936 for whole transcriptome RT settings, P < 0.001). In other words, the better RT priming method (gene-specific) resulted in a larger improvement of RT-qPCR sensitivity than the better RNA isolation method (Qiagen). The total average improvement in sensitivity between the workflows using either Qiagen extraction combined with gene-specific priming or Epicentre extraction combined with whole transcriptome priming was 7.21 fold (∆Cq = 2.85, 95% CI = 2.58–3.12, P < 0.001).

The signal in the NTC sample cluster is significantly lower (higher Cq values) than the signal in the other clusters (average Cq = 32.4, CI = 31.3–33.4, P < 0.001, Fig. 5b). If only the no template control cluster is considered, the difference of non-specific amplification signal magnitude between all NTC samples is insignificant (P > 0.1). The occurrence of non-target-specific amplification signal was observed in all 4 tested NTC conditions.

## Discussion

Although the importance of FFPE-tailored RNA isolation methods is well documented^14^, we decided to use two commercially available RNA isolation kits to determine the effect of RNA quality on qPCR sensitivity. Both tested kits include a proteinase K digestion and heat treatment, which are considered as key factors for reverse formaldehyde crosslinking and recovery of more intact RNA^13^,^15^,^16^. Yet, a significant difference was observed between the tested RNA isolation methods. The column-based RNA isolation method (Qiagen) allowed recovery of 3.25 times more RNA and showed consistently higher RNA integrity in 4 cell pellet FFPE samples and 20 tumor FFPE samples. As the same amount of RNA was used for downstream analysis, only the RNA integrity impacted RT-qPCR sensitivity. RNA samples with better quality allowed earlier detection by a modest average of 0.7 PCR cycles, for cell line pellets and tissue FFPE samples. This suggests that better RNA quality from FFPE samples can only improve RT-qPCR sensitivity to a limited extent. This finding can be attributed to the presence of residual chemical modifications on RNA molecules, despite proteinase K and heat treatment. Primer annealing and elongation is thought to be inefficient due to the presence of these chemical alterations^13^. We therefore believe that, unless new procedures are developed to efficiently undo these chemical modifications, RNA from FFPE samples is likely to remain a suboptimal substrate for RT-qPCR analysis.

The age of FFPE blocks used in this study ranges from 3 to 6 year. Even though high degradation was observed for the vast majority of the samples, the RNA isolation method may result in different degradation profiles for older FFPE samples. However, the profiles we obtained from capillary electrophoresis, revealing severe fragmentation and enrichment in RNA fragments below 200 nucleotides, are typically observed in FFPE samples in general, including samples older than 10 or 20 years^10^,^23^.

Since improved RNA quality did not increase RT-qPCR sensitivity to a large extent, we decided to further evaluate two downstream RT-qPCR steps: reverse transcription and preamplification.

First, multiplex gene-specific reverse transcription was tested. To that purpose, 48 qPCR primers, complementary to the target RNA sequences, were pooled and gene-specific reverse transcription was performed. A similar but limited experiment was performed by Nardon *et al*., in which a four-plex gene-specific priming was shown to be more sensitive than oligo-dT or random priming in FFPE material^24^. In our study, 48–plex priming was tested in duplicate RT reactions and duplicate qPCR reactions on 8 FFPE RNA samples isolated by 2 different methods. An average 4.0 fold increase in RT-qPCR sensitivity was observed in RNA samples obtained by the Qiagen kit. The improvement was at least as high in RNA samples of lower quality (an average 4.9 fold increase in RT-qPCR sensitivity), suggesting that gene-specific reverse transcription reaction was more powerful than higher RNA quality to raise the sensitivity of gene expression measurements in FFPE material.

Of note, the qPCR assays used in this study generally produced short amplicons (Supplemental Table 3), which is well documented to improve PCR amplification if RNA is fragmented or degraded; such assays have definitely contributed to the successful gene expression measurements using FFPE samples in this study.

In the following step, multiplex gene-specific preamplification was tested. A drastic increase in RT-qPCR sensitivity was obtained as preamplification enabled earlier gene detection, shifting the results towards lower Cq values by approximately 7 cycles in both fresh frozen and in FFPE samples, substantially decreasing the number of non-detected genes.

Previous studies have evaluated preamplification of FFPE RNA samples. These studies usually apply probe based qPCR assay detection technology (likely to avoid detection of non-specific by-products), with premanufactured gene-specific RT pools and preamplification assay pools^25^,^26^. In our study, custom multiplex gene-specific preamplification was performed starting from a universal reverse transcription reaction (mixed oligo-dT and random priming). This not only avoids reverse transcription mediated non-specific product formation^27^, but also simplifies the workflow and reduces costs and hands-on time.

Only a few studies have tested multiplex gene-specific preamplification on whole transcriptome RT product^21^,^22^. For example, Li *et al.* (2008) pooled 8 TaqMan preamp assays to test preamplification on 8 FFPE RNA samples^19^. For evaluation of gene expression linearity between preamplified and non-preamplified samples, the gene expression difference was calculated for only one gene across 8 samples. While comparable differences were obtained for the CDKNIB gene in preamplified and non-preamplified samples, the significance of these findings was not determined. Another more comprehensive study, also involving TaqMan preamplification on universal RT samples used TaqMan low-density array microfluidics cards to measure expression of 96 genes in preamplified and non-preamplified RNA from FFPE material^28^. Expression differences between two samples were calculated and Spearman correlation values of 0.89, 0.90 and 0.84 were found between preamplified and non-preamplified samples, in normal fresh frozen tissue, normal breast FFPE tissue and tumor breast FFPE tissue, respectively.

In our study, very similar results were observed. Pearson correlation values of at least 0.99 and 0.86 were found between preamplified and non-preamplified, in fresh frozen material and FFPE material respectively. We also calculated Pearson correlation between preamplified FFPE samples and matched non-preamplified fresh frozen samples (Table 1). Equally high correlation values were observed (Table 1). Of note, we do not recommend comparison of gene expression between samples of different quality. We previously discussed the importance of using similar RNA quality when performing RT-qPCR- based gene-expression studies^12^.

An increased non-specific product formation was observed in no template control RT reactions that underwent preamplification (compared to negative control PCR reactions using water only). An average Cq-value of 31.9 was measured in these negative control samples, which is below the Cq cutoff of 35 cycles chosen for this study. Therefore, we recommend adjustment of the detection cutoff towards lower Cq values (32 or 30 cycles) when preamplification is used and careful evaluation of melting curve profiles when low expressed genes (high Cq values) are being investigated.

Finally, cluster analysis, performed on non-normalized Cq values from all the workflows, was used to visualize differences between preamplification, gene-specific reverse transcription and whole transcriptome reverse transcription (Fig. 4). Preamplification resulted in the most sensitive RT-qPCR data, uniformly across all tested genes. Gene-specific reverse transcription was the second best method and we showed that gene-specific reverse transcription had a larger effect on qPCR sensitivity than RNA quality. Whole transcriptome RT without preamplification was found to be the least sensitive method for RNA expression analysis of FFPE samples.

To the best of our knowledge, there are no studies that have systematically quantified the effect of RT-qPCR workflow adjustments on the final gene detection sensitivity. We showed that increased RNA quality only improves qPCR sensitivity to a limited extent and that gene-specific reverse transcription and gene-specific preamplification may be more useful for RNA expression studies in FFPE material. Many studies engaged with validation of multi-gene expression classifiers in FFPE sample cohorts are confronted with compromised RT-qPCR sensitivity, shifts towards high (and variable) Cq values and high occurrence of missing data^29^. This interferes with the performance of gene-expression classifiers in FFPE material as not all genes of interest may be detected^20^,^30^. Therefore, implementation of gene-specific preamplification or gene-specific reverse transcription may considerably increase the success rate of validation of molecular classifiers in FFPE sample cohorts.

## Materials and Methods

### Sample preparation

Four cell lines (HEK-293T, NGP, MCF7, SK-N-AS) were cultured to subconfluency in T75 flasks, harvested by scraping, collected in 15 ml tubes and fixed and embedded according to standardized operation procedures at the Department of Pathological Anatomy (Ghent University Hospital, Belgium). FFPE cell pellets were prepared using a semi-automated protocol, composed of two main steps: overnight fixation at 4% formalin (Klinipath), tissue dehydration with 96% ethanol and xylene treatment, immediately followed by paraffin embedding (Histowax) on the Tissue-TEK VIP device (Sakura Finetek). From each FFPE block, a series of 20 μM sections were cut.

For validation tissue FFPE samples, 10 μM sections from anonymized tumor blocks were used (prepared between 2007 and 2010). Negative control samples consisted of MS2-phage RNA diluted in RNase-free water (50 ng/μl).

### RNA isolation

Two kits were used for RNA isolation: miRNeasy FFPE kit (Qiagen) and Master Pure RNA Purification kit (Epicentre). Three parallel RNA isolations were performed from each cell pellet FFPE block in order to obtain sufficient RNA for the downstream qPCR analyses: two isolation rounds were performed on five 20 μM FFPE sections and the third on eight 20 μM FFPE sections. One 10 μM section was used for RNA isolation from FFPE tumor samples.

RNA isolation with RNeasy FFPE kit (Qiagen) was performed according to the manufacturer’s guidelines. Briefly, each sample was mixed with buffer PKD followed by Proteinase K digestion and heat treatment (incubation at 56 °C for 15 minutes and at 80 °C for 15 minutes). Centrifugation at 20,000 g for 15 minutes allowed separation of the DNA/RNA containing phase. DNase treatment was applied for genomic DNA removal. Each sample was then mixed with ethanol for subsequent transfer to RNeasy MinElute spin columns. The columns were centrifuged at 8,000 g for 15 seconds. This step allowed capturing of RNA in the porous membrane and removal of the supernatant. The membrane was washed twice with RPE buffer and dried by spinning the unclosed tubes. The RNA was released from the membrane with 30 μl RNase-free water.

RNA isolation with Master Pure RNA purification kit (Epicentre) was performed according to the manufacturer’s guidelines. Briefly, each sample was treated with proteinase K and incubated at 65 °C for 30 minutes. Total nucleic acid was precipitated and the pellet was washed with isopropanol and treated with DNase for genomic DNA removal followed by precipitation of gDNA-free RNA. The purified RNA was rinsed twice with ethanol and resuspended in 30 μl TE buffer.

### RNA quality assessment

RNA integrity was evaluated by capillary electrophoresis (Experion RNA StdSens Analysis kit, Bio-Rad) according to the manufacturer’s protocol. Briefly, denatured RNA samples were transferred on RNA StdSens chips, which were primed with a mixture of gel-stain solution and loading buffer. The samples were run on Experion electrophoresis station. The data was analyzed by evaluation of virtual gel reports with the Experion software (Supplemental Fig. 1).

RNA concentrations were measured by spectrophotometry on NanoDrop1000 (Supplemental Table 1 and 2).

Genomic DNA contamination was evaluated by performing qPCR using primers targeting the (non-expressed) promoter regions of *NEUROD1* and *XRCC3* (primer sequences in Supplemental Table 3)^31^. Each 5 μl reaction contained 2.5 μl 2× SsoAdvanced SYBR Green Universal Supermix, 250 nM (final concentration) of each forward and reverse primer and 2 μl of diluted RNA solution (see below). Cq-values obtained from the RNA-samples were compared to Cq values obtained from negative control samples spiked with 0.5 ng genomic DNA (Roche Human Genomic DNA). 8 randomly selected RNA FFPE samples isolated with Qiagen and 8 RNA FFPE samples isolated with Epicentre were tested.

### qPCR assays

Primers were designed using primerXL (http://www.primerxl.org) and synthesized by Integrated DNA Technologies (IDT) as standard desalted primers. The target list contains genes that are expressed in both human brain and universal reference RNA^32^. Primers sequence, RTPrimerDB ID, PCR efficiency and specificity are shown in Supplemental Table 4. RNase-free water was added to lyophilized primers to obtain a 250 μM stock concentration, stored at −20 °C. Primers were diluted to 5 μM use concentration and 0.25 μl is used in a 5 μl qPCR reaction (250 nM final concentration).

### Reverse transcription

iScript Select cDNA Synthesis kit was used for **multiplex gene-specific reverse transcription**, according to the manufacturer’s guidelines. Briefly, 20 μl reactions were prepared by combining 4 μl iScript Select reaction mix, 2 μl gene-specific enhancer solution, 1 μl reverse transcriptase, 1 μl gene-specific assay pool (20×, 2 μM), 12 μl RNA diluted in RNase-free water. For preparation of the gene-specific assay pool only primers complementary to the RNA sequence (the antisense primer) were used. 48 antisense primers were pooled and diluted to a final 2 μM concentration per primer. The final concentration of each primer in the 20 μl RT reaction was 100 nM. The reverse transcription reactions were performed by incubating for 30 minutes at 42 °C and 5 minutes at 85 °C.

iScript cDNA synthesis kit was used for ***whole transcriptome reverse transcription***, according to the manufacturer’s guidelines. Briefly, 20 μl reactions were prepared by combining 4 μl iScript reaction mix (containing a blend of random and oligo-dT primers), 2 μl RNA, 1 μl reverse transcriptase, and 13 μl RNase-free water. The RT reaction was performed by incubating for 5 minutes at 25 °C, followed by 30 minutes at 42 °C and 5 minutes at 85 °C.

The amount of total RNA input varies in different sections of the study (Supplemental Table 5). 300 ng of total RNA was used for comparison of multiplex gene-specific RT to whole transcriptome RT in cell pellet FFPE samples. 600 ng of total RNA was used for comparison of multiplex gene-specific RT to whole transcriptome RT in tumor FFPE samples. 600 ng of total RNA was used to compare gene-expression ratios in non-preamplified (whole transcriptome RT) samples between matched FFPE and fresh frozen material and 50 ng of total RNA was used to compare gene expression ratios in preamplified samples between matched FFPE and fresh frozen material.

Each reverse transcription product was diluted 15-fold by adding 280 μl H2O to 20 μl cDNA.

### Sample preamplification

For multiplex gene-specific pre-amplification, cDNA from whole transcriptome cDNA synthesis was used. SsoAdvanced PreAmp Supermix (Bio-Rad) was used for 48-plex preamplification reactions. 10 μl undiluted RT was added to 12.5 μl 2× SsoAdvanced Preamp Supermix and 2.5 μl custom-made PreAmp assay pool (10x, 500 nM). For the PreAmp assay pool, 96 primers were pooled (forward and reverse primer from 48 assays) to obtain a 500 nM concentration for each primer. The final primer concentration in the preamplification reaction was 50 nM. The preamplification reaction consisted of an activation step at 98 °C for 3 minutes and a two-step 12-cycle amplification: 98 °C for 15 seconds and 58 °C for 4 minutes. The preamplified cDNA was diluted 20-fold by adding 475 μl H2O to 20 μl cDNA.

### Quantitative PCR

SsoAdvanced SYBR Green Universal Supermix (Bio-Rad) was used for 5 μl qPCR reaction. Reactions were run in duplicate in 384-multiwell plates (Bio-Rad). Each 5 μl reaction contained 2.5 μl SsoAdvanced SYBR Green Universal Supermix, 250 nM of each forward and reverse primer and 2 μl of 15-fold diluted cDNA or 20-fold diluted preamp product. The qPCR reactions were run on CFX384 instrument. The thermal cycling protocol starts with polymerase activation at 95 °C for 30 seconds, followed by 40 cycles of denaturation at 98 °C for 15 seconds, annealing/extension and read out at 60 °C and ends with melt curve analysis during 5 second 0.5 °C increment steps from 65 °C to 95 °C. qPCR runs were performed on the LightCycler480 instrument (Roche). Cq values were determined based on the maximum of the second derivative of the amplification curve by the LightCycler480 software version 1.5.0 SP4.

### Study design and data analysis

In this study, three different RT-qPCR protocols were tested for gene expression analysis of FFPE RNA samples (Fig. 1). In the first and the second protocol, whole transcriptome reverse transcription was compared to multiplex gene-specific reverse transcription. Qiagen RNA and Epicentre RNA were used for the first and the second protocol, respectively. The effect of multiplex gene-specific preamplification was tested in the third protocol where only Epicentre RNA samples (lowest RNA integrity) were used. Throughout the study, duplicate RT reactions and duplicate qPCR reactions were used to evaluate gene expression in cell pellet FFPE samples. Single RT reactions followed by duplicate qPCR reactions were used for evaluation in tumor tissue FFPE samples.

Data analysis was performed on non-normalized Cq values. Cq detection limit was set at 35, unless otherwise mentioned in the results section. Expression difference refers to Cq difference (∆Cq) between two samples. Earlier detection refers to lower Cq values or higher gene expression. Fold increase in RT-qPCR sensitivity is calculated as 2 to the power of ∆Cq (2^∆Cq^), with ∆Cq being the difference in Cq between 2 conditions.

## Additional Information

**How to cite this article**: Zeka, F. *et al.* Straightforward and sensitive RT-qPCR based gene expression analysis of FFPE samples. *Sci. Rep.* **6**, 21418; doi: 10.1038/srep21418 (2016).

## Supplementary Material

Supplementary Information

## Figures and Tables

**Table 1:** Pearson correlations coefficients for qPCR cycle difference (∆Cq) between preamplified and non-preamplified samples.

∆Cq	FF-FF	FFPE-FFPE	FFPE-FF
NGP – HEK293	0.996	0.867	0.886
NGP – SK-N-AS	0.992	0.885	0.902
NGP – MCF7	0.995	0.886	0.899
HEK293 – SK-N-AS	0.995	0.940	0.931
HEK293 – MCF7	0.997	0.935	0.874
SK-N-AS – MCF7	0.996	0.960	0.916

All P-values for Pearson correlations < 0.001; correlations calculated for 48 genes.

**Table 2:** Overview of RT-qPCR conditions performed on HEK-293T FFPE cell pellets and no template control samples.

cell line	whole transcriptome RT*	gene-specific RT*	gene-specific PA**	Qiagen	Epicentre
HEK	+			+	
HEK	+			+	
HEK	+				+
HEK	+				+
HEK		+		+	
HEK		+		+	
HEK		+			+
HEK		+			+
HEK	+		+		+
HEK	+		+		+
PA NTC	+		+		
PA + MS2 NTC	+		+		
WT NTC	+				
WT NTC	+				
GSP NTC		+			
GSP NTC		+			
H2O NTC***					
H2O NTC***					

RT = reverse transcription; WT = whole transcriptome; GSP = gene specific; PA = preamplification; Q = Qiagen; E = Epicentre.

HEK = HEK-293T FFPE cell pellet RNA; MS2 = MS2 phage carrier RNA.

(*) 300 ng RNA was used for whole transcriptome and gene-specific RT reactions; (**) 50 ng RNA was used for whole transcriptome RT reactions intended for preamplification; (***) H2O NTC samples were only used for 5 μl qPCR reactions.

## References

[b1] Wong M. L. & Medrano J. F. Real-time PCR for mRNA quantitation. Biotech. 1–11 (2005). 10.2144/05391RV01 16060372

[b2] Tournoud M. *et al.* A strategy to build and validate a prognostic biomarker model based on RT-qPCR gene expression and clinical covariates. BMC Bioinformatics 16, 3401 (2015). 10.1186/s12859-015-0537-9 PMC4384357 25880752

[b3] Vermeulen J. *et al.* Predicting outcomes for children with neuroblastoma using a multigene-expression signature: a retrospective SIOPEN/COG/GPOH study. Lancet Oncology 10, 663–671 (2009). 19515614 10.1016/S1470-2045(09)70154-8 PMC3045079

[b4] De Preter K. *et al.* miRNA Expression profiling enables risk stratification in archived and fresh neuroblastoma tumor samples. Clinical Cancer Research 17, 7684–7692 (2011). 22031095 10.1158/1078-0432.CCR-11-0610 PMC4008338

[b5] Tanney A. & Kennedy R. D. Developing mRNA-based biomarkers from formalin-fixed paraffin-embedded tissue. Personalized Medicine 7, 205–211 (2010). 10.2217/pme.10.8 29783318

[b6] Klein D. Quantification using real-time PCR technology: applications and limitations. Trends in Molecular Medicine 1–4 (2002). 12067606 10.1016/s1471-4914(02)02355-9

[b7] Kong H. *et al.* Quantitative assessment of short amplicons in FFPE-derived long-chain RNA. Sci. Rep. 4, 7246 (2014). 25430878 10.1038/srep07246 PMC5384205

[b8] Greytak S. R., Engel K. B., Bass B. P. & Moore H. M. Accuracy of molecular data generated with FFPE biospecimens: lessons from the literature. Cancer Research 75, 1541–1547 (2015). 25836717 10.1158/0008-5472.CAN-14-2378 PMC4636024

[b9] Xie R. *et al.* Factors influencing the degradation of archival formalin-fixed paraffin-embedded tissue sections. Journal of Histochemistry & Cytochemistry 59, 356–365 (2011). 21411807 10.1369/0022155411398488 PMC3201147

[b10] Cronin M. *et al.* Measurement of gene expression in archival paraffin-embedded tissues: development and performance of a 92-gene reverse transcriptase-polymerase chain reaction assay. The American Journal of Pathology 164, 35–42 (2004). 14695316 10.1016/S0002-9440(10)63093-3 PMC1602211

[b11] Bustin S. A. *et al.* The MIQE Guidelines: Minimum Information for Publication of Quantitative real-time PCR experiments. Clinical Chemistry 55, 611–622 (2009). 19246619 10.1373/clinchem.2008.112797

[b12] Vermeulen J. *et al.* Measurable impact of RNA quality on gene expression results from quantitative PCR. Nucleic Acids Research 39, e63–e63 (2011). 21317187 10.1093/nar/gkr065 PMC3089491

[b13] Masuda N., Ohnishi T., Kawamoto S., Monden M. & Okubo K. Analysis of chemical modification of RNA from formalin-fixed samples and optimizationof molecular biology applications for such samples. Nucleic Acids Research 1–8 (1999). 10536153 10.1093/nar/27.22.4436 PMC148727

[b14] Ahlfen von S., Missel A., Bendrat K. & Schlumpberger M. Determinants of RNA quality from FFPE samples. PLoS ONE 2, e1261 (2007). 18060057 10.1371/journal.pone.0001261 PMC2092395

[b15] Chung J.-Y. *et al.* Optimization of recovery of rna from formalin-fixed, paraffin-embedded tissue. Diagn. Mol. Pathol. 1–8 (2006). 17122651 10.1097/01.pdm.0000213468.91139.2d

[b16] Benchekroun M. *et al.* Impact of fixative on recovery of mRNA from paraffin-embedded tissue. Diagn. Mol. Pathol. 13, 116–125 (2004). 15167013 10.1097/00019606-200406000-00008

[b17] Zhao W. *et al.* Comparison of RNA-Seq by poly (A) capture, ribosomal RNA depletion, and DNA microarray for expression profiling. BMC Genomics 15, 1–11 (2014). 24888378 10.1186/1471-2164-15-419 PMC4070569

[b18] Specht K. *et al.* Quantitative gene expression analysis in microdissected archival formalin-fixed and paraffin- embedded tumor tissue. American Journal of Pathology 1–11 (2001). 11159180 10.1016/S0002-9440(10)63985-5 PMC1850313

[b19] Sánchez-Navarro I. *et al.* Comparison of gene expression profiling by reverse transcription quantitative PCR between fresh frozen and formalin-fixed, paraffin-embedded breast cancer tissues. Biotech. 48, 389–397 (2010). 10.2144/000113388 20569212

[b20] Denning K. M. *et al.* A molecular expression signature distinguishing follicular lesions in thyroid carcinoma using preamplification RT-PCR in archival samples. Mod Pathol 20, 1095–1102 (2007). 17660800 10.1038/modpathol.3800943

[b21] Li J. *et al.* Improved RNA quality and TaqMan^®^ Pre-amplification method (PreAmp) to enhance expression analysis from formalin fixed paraffin embedded (FFPE) materials. BMC Biotechnol 8, 10 (2008). 18254955 10.1186/1472-6750-8-10 PMC2259333

[b22] Vera-Lozada G. *et al.* Analysis of biological and technical variability in gene expression assays from formalin-fixed paraffin-embedded classical Hodgkin lymphomas. Experimental and Molecular Pathology 97, 433–439 (2014). 25236575 10.1016/j.yexmp.2014.09.014

[b23] Hewitt S. M. *et al.* Tissue handling and specimen preparation in surgical pathology. 1–7 (2008). 10.5858/132.12.1929 19061293

[b24] Nardon E., Donada M., Bonin S., Dotti I. & Stanta G. Higher random oligo concentration improves reverse transcription yield of cDNA from bioptic tissues and quantitative RT-PCR reliability. Experimental and Molecular Pathology 87, 146–151 (2009). 19619529 10.1016/j.yexmp.2009.07.005

[b25] Li P. *et al.* Evaluation of a high-throughput, microfluidics platform for performing TaqMan™ qPCR using formalin-fixed paraffin-embedded tumors. Bioanalysis 5, 1623–1633 (2013). 23822126 10.4155/bio.13.125 PMC3816109

[b26] Yeo J. *et al.* A Multiplex two-color real-time PCR method for quality-controlled molecular diagnostic testing of FFPE samples. PLoS ONE 9, e89395 (2014). 24586747 10.1371/journal.pone.0089395 PMC3931751

[b27] Vandesompele J., De Paepe A. & Speleman F. Elimination of primer–dimer artifacts and genomic coamplification using a two-step SYBR Green I real-time RT-PCR. Analytical Biochemistry 303, 95–98 (2002). 11906156 10.1006/abio.2001.5564

[b28] Ciotti P. *et al.* Reliability and reproducibility of a RNA preamplification method for low-density array analysis from formalin-fixed paraffin-embedded breast cancer samples. Diagn. Mol. Pathol. 18, 112–118 (2009). 19430292 10.1097/PDM.0b013e3181831320

[b29] Antonov J. *et al.* Reliable gene expression measurements from degraded RNA by quantitative real-time PCR depend on short amplicons and a proper normalization. Lab Invest 85, 1040–1050 (2005). 15951835 10.1038/labinvest.3700303

[b30] Nishio M. *et al.* 72-Gene classifier for predicting prognosis of estrogen receptor-positive and node-negative breast cancer patients using formalin-fixed, paraffin-embedded tumor tissues. Clinical Breast Cancer 14, e73–e80 (2014). 24461457 10.1016/j.clbc.2013.11.006

[b31] Vermeulen J. *et al.* RNA pre-amplification enables large-scale RT-qPCR gene-expression studies on limiting sample amounts. BMC Res Notes (2009). 10.1186/1756-0500-2-235 PMC2789097 19930725

[b32] Shi L. *et al.* The MicroArray Quality Control (MAQC) project shows inter- and intraplatform reproducibility of gene expression measurements. Nat Biotechnol 24, 1151–1161 (2006). 16964229 10.1038/nbt1239 PMC3272078

