# Supplementary material for: Straightforward and sensitive RT-qPCR based gene expression analysis of FFPE samples
Source: Sci Rep. 2016 Feb 22;6:21418. doi: 10.1038/srep21418 (PMC4761903; doi:10.1038/srep21418)
Supplement: Supplementary Information [file srep21418-s1.pdf]

Straightforward and sensitive RT-qPCR based gene expression analysis of FFPE samples

Fjoralba Zeka*[1,2], Katrien Vanderheyden[1,2], Els De Smet[1,2], Claude Cuvelier[3], Pieter Mestdagh[1,2], Jo Vandesompele[1,2]

[1] Center for Medical Genetics, Ghent University, Belgium

[2] Cancer Research Institute Ghent, Ghent University, Belgium

[3] Department of Pathological Anatomy, Ghent University Hospital, Belgium

*Corresponding author: Jo Vandesompele (joke.vandesompele@ugent.be)

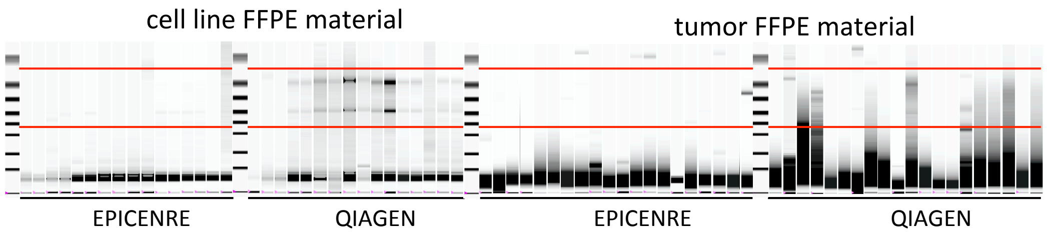

Supplemental Figure 1: Microfluidic electrophoresis lanes obtained by Experion for paired Qiagen-Epicentre RNA samples prepared from 16 cell line FFPE samples and 20 tumor tissue FFPE samples. Ribosomal bands are shown within the region marked by red lines.

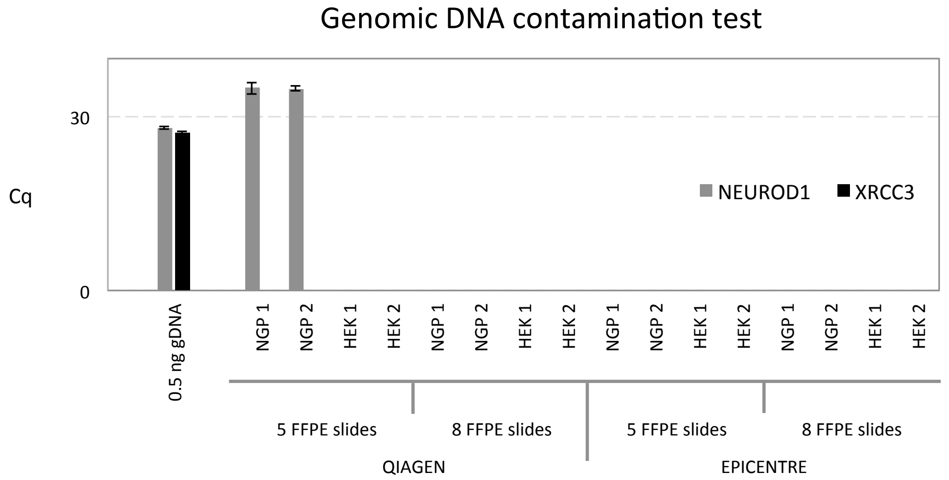

Supplemental Figure 2: Amplification of NEUROD1 and XRCC3 in presence of 0.5 ng Human Genomic DNA and 3 µl total RNA extract isolated with Qiagen or Epicentre protocol, from 5 FFPE slides and 8 FFPE slides for the given cell lines

Supplemental Table 1: RNA concentrations (ng/µl) measured by NanoDrop 1000 for RNA samples isolated with the protocol provided by QIAGEN and the protocol provided by Epicentre for cell pellet FFPE

	Qiagen (ng/µl)				Epicentre (ng/µl)			
# sections	HEK	NGP	SKNAS	MCF7	HEK	NGP	SKNAS	MCF7
5	296.1	81.5	123.3	93.1	172.6	41.4	85.1	73.6
5	555.1	70.0	122.6	72.0	150.5	48.4	73.5	47.2
5	104.9	39.2			85.7	38.5		
5	92.3	11.1			87.7	40.4		
8	291.7	68.4			84.3	30.4		
8	667.3	37.7			79.8	27.3		

Supplemental Table 2: RNA concentrations (ng/µl) measured by NanoDrop 100 for 20 FFPE tumor samples isolated with the protocol provided by QIAGEN and the protocol provided by Epicentre

Qiagen (ng/µl)	Epicentre (ng/µl)	difference	sample reference to Supplemental Figure 1
82.7	45.9	36.7	T1
450.1	245.5	204.7	T2
501.5	47.2	454.2	T3
311.7	44.9	266.8	T4
975.3	141.8	833.6	T5
381.6	37.7	343.9	T6
270.3	76.5	193.8	T7
13.4	69.7	-56.3	T8
967.9	71.4	896.6	T9
155.1	57.8	97.3	T10
665.5	370.4	295.1	T11
972.6	385.8	586.8	T12
1332.1	238.5	1093.6	T13
55.3	297.6	-242.2	T14
538.1	94.9	443.2	T15
1347.8	501.6	846.1	T16
493.8	382.8	110.9	T17
300.9	605.4	-304.5	T18
886.8	1318.5	-431.7	T19
790.3	198.3	592.0	T20

Supplemental Table 3: RT-qPCR primer properties for genomic DNA contamination testing

target ID	refseq ID	forward primer	reverse primer	annealing temp	efficiency	amplicon length (nt)	RTprimer DB ID	template
NEUROD1	NG_011820.1	CTTCTGCCGCCTGAAAGG	CCTGGAACCACGTGACCTG	60	85-115%	154	8113	DNA (promotor region)
XRCC3	NG_011516	CTTGATTCTTTCTAGCCTTGG	GGTTGACACTTTGATGGATAC	60	85-115%	239	-	DNA (promotor region)

Table 4: RT-qPCR primer properties

target ID	refseq ID	forward primer	reverse primer	annealing temp	efficiency	melt curve	amplicon length	RTprimer DB ID
ABCB6	NM_005689	ACATTGTGAACTTGCTGACT	AGGAACTTGAGGAAGACGTA	60	85% - 90%	single peak	82	7903
ABCG2	NM_004827	TGACCTGAAGGCATTTACTG	GGTAGAAAGCCACTCTTCAG	60	90% - 95%	single peak	84	7905
ADM	NM_001124	AGACTGATTACCTCCTGTGT	TGTTCGCATATCACCCATTT	60	90% - 95%	single peak	82	7913
ALDH4A1	NM_003748	GTGTTCTCCCAGGATAAGGA	GTGGACTTGTCGTTGATGTA	60	< 85%	single peak	89	7916
ALKBH1	NM_006020	TCAGTCCAAGGAGGTATGTT	ACTCTGCAAACATGGAAACT	60	< 85%	single peak	94	7917
ANXA1	NM_000700	TGGATGAAGCAACCATCATT	CCTGTTTCCTGGAGATATGC	60	90% - 95%	single peak	88	7919
ANXA5	NM_001154	TGAACTGAAACATGCCTTGA	GATGGCTCTCAGTTCTTCAG	60	90% - 95%	single peak	94	7920
AP4B1	NM_006594	CTGGTGAACGATGAGAATGT	GACCCAGCAACTCTGTTAAA	60	85% - 90%	single peak	160	7921
ATG3	NM_022488	GCCGTTAAAGAGATCACACT	CATATCTGCAGCTTCTCCTT	60	90% - 95%	single peak	108	7926
ATP6AP1	NM_001183	TTCTGGAATGACTCCTTTGC	TTGGCCAGAATGAACTTGAA	60	90% - 95%	single peak	86	7927
CRLF1	NM_004750	CCCTCAAGTACAAGCTTAGG	CCAGATCTCATAGGGCGTAA	60	85% - 90%	single peak	122	7949
CUEDC2	NM_024040	CTGCAGAAGTACATGATGGT	ACTACCTGGTTGTCGATGTA	60	85% - 90%	single peak	110	7952
DCXR	NM_016286	TCCGAGTGAATGCAGTAAAC	TTCGGTTCAGCATAGTCTTG	60	90% - 95%	single peak	96	7954
DDRGK1	NM_023935	GCATGAGGAGTACCTGAAAC	TGGACTGCTTGATGTAGTTG	60	90% - 95%	single peak	125	7955
DNAJB1	NM_006145	TGCTCAGACCTTTAGACTCA	CTGTCGCACTTCATTGACTA	60	90% - 95%	single peak	81	7958
DNAJC7	NM_001144766	GGAGAGGCCTTTACTATCCT	ATTCATGCCCTCCTCATCTA	60	90% - 95%	single peak	84	7960
ELK1	NM_001114123	GAAGAATCACACCCTTGGAA	GACAAAGGAATGGCTTCTCA	60	85% - 90%	single peak	135	7963
ERCC3	NM_000122	TCCGGAATGATTCTGTCAAC	GCAAGCTCTTCTCCTGATAG	60	90% - 95%	single peak	84	7964
FANCG	NM_004629	TAGTTGAGGCCTTGAATGTC	CTTGCTAGTATGTGCTTGGT	60	90% - 95%	single peak	139	7965
HSP90B1	NM_003299	CCGAAGAAGAACCTGAAGAG	CATCTGTTCCCACATCCATT	60	90% - 95%	single peak	87	7975
JUN	NM_002228	ACAGCTTCATGCCTTTGTAA	CTCAGAGTGCTCCAAATCTC	60	90% - 95%	single peak	93	7981
LDLR	NM_000527	AACTGCCATTGTCGTCTTTA	ACATACCCATCAACGACAAG	60	90% - 95%	single peak	102	7986
LMBRD1	NM_018368	TAAGGACTGGGCTAATGCTA	CCAGAAGAACACACAGAACA	60	90% - 95%	single peak	103	7988
LXN	NM_020169	ATGACGCTCGTTCTACATTT	TTTCTTTGCACTTGCTTGAC	60	85% - 90%	single peak	119	7990
MAP3K5	NM_005923	CACTGAATGTACAGCTTGGA	CGATGAAGGAGTGCTTGTAA	60	90% - 95%	single peak	97	7991
MBOAT7	NM_001146056	GGGCAAATTCCCTTTCTTTG	TATTGGTGCGATGAGCTAAA	60	90% - 95%	single peak	117	7993
MCM3	NM_002388	CCACAGATGATCCCAACTTT	GTCCCATGTAGAAGGTTGTC	60	90% - 95%	single peak	82	7994
MGMT	NM_002412	ATTAAAGGAAGTGGCAGTGT	CAGCGTTAGAGAAGGAAACA	60	90% - 95%	single peak	122	7999
MRPL13	NM_014078	TGGACTCCACCTGAAGATTA	AAACAGGTGCTGAACTGTAG	60	90% - 95%	single peak	140	8001
MYO5A	NM_000259	GCCTCAAGTTCCTCGTTTAT	CTAAGGGCTGAGAAGAGAGA	60	90% - 95%	single peak	91	8004
NME1	NM_000269	TCATTGCGATCAAACCAGAT	CAACGTAGTGTTCCTTGAGA	60	90% - 95%	single peak	138	8007
P4HB	NM_000918	GCAAACTGAGCAACTTCAAA	TTCTTCAGGCCAAAGAACTC	60	90% - 95%	single peak	118	8013
PAICS	NM_001079524	GGATGTGTGGTCTTCTCTTC	AGCAAATTGAGCTGATCCTT	60	90% - 95%	single peak	82	8015
PKIA	NM_006823	TGGTAGCAATGACTGATGTG	GCATCTTCTTCACCTTCTGT	60	90% - 95%	single peak	175	8021
PPARD	NM_006238	TGTCTCCCTCTTTCTCAGTT	AGGGAAGAGAGAAACCTACA	60	90% - 95%	single peak	82	8025
PPIC	NM_000943	TAGCAACAGGAGAGAAAGGA	GTGATGTCACCTCCTTGAAT	60	90% - 95%	single peak	88	8026
PPP3CA	NM_000944	TGTGATATCCTGTGGTCAGA	CTGACTGTGTTGTGAGTGAA	60	90% - 95%	single peak	80	8028
PSMB10	NM_002801	CAGACAGTGAAGCCACTAAC	CTCTAAGCCTCAGCTTACTC	60	85% - 90%	single peak	80	8032
RSAD1	NM_018346	GGAGAGAATTCGGGAAAGAC	CAGGCTCCAGATGTGTTTAT	60	90% - 95%	single peak	112	8042
SERPINI1	NM_001122752	ACTGTTCTGGGAGATCTGAA	CAGCAAAGAGAAGAGTCCAA	60	90% - 95%	single peak	127	8047
SYNGR1	NM_004711	TCCTAAGCAGAGAAGTAGCA	TGGACACACCTGAGTTTAGA	60	90% - 95%	single peak	111	8062
TNFAIP1	NM_021137	TTTCCAAAGGAATGCTGTCA	AGAGCAGAAAGTGGGTACTA	60	< 85%	single peak	85	8072
TTC15	NM_016030	AGTTTGGATCCGAAGAGAAC	CTCCATCATGTGTTCGTTCA	60	85% - 90%	single peak	80	8076
VAMP3	NM_004781	TCCCAGTTAGTTACCCTTGT	CTGGAAGGCATAAGTTGGAA	60	90% - 95%	single peak	90	8081
SDHA	NM_004168.3	TGGGAACAAGAGGGCATCTG	CCACCACTGCATCAAATTCATG	60	85%-115%	single peak	86	8094
HMBS	NM_000190.3	GGCAATGCGGCTGCAA	GGGTACCCACGCGAATCAC	60	85%-115%	single peak	64	8095
HPRT1	NM_000194.2	TGACACTGGCAAAACAATGCA	GGTCCTTTTCACCAGCAAGCT	60	85%-115%	single peak	94	8097
YWHAZ	NM_003406	ACTTTTGGTACATTGTGGCTTCAA	CCGCCAGGACAAACCAGTAT	60	85%-115%	single peak	84	8100

Supplemental Table 5: overview RT-qPCR conditions performed on cell pellet FFPE samples

cell line	experimental purpose	RNA input in RT reaction (ng)	whole genome RT	gene-specific RT	gene-specific PA	Qiagen	Epicentre
HEK	WT vs. GSP	300	+			+	
HEK	WT vs. GSP	300	+			+	
HEK	WT vs. GSP	300	+				+
HEK	WT vs. GSP	300	+				+
HEK	WT vs. GSP	300		+		+	
HEK	WT vs. GSP	300		+		+	
HEK	WT vs. GSP	300		+			+
HEK	WT vs. GSP	300		+			+
Tumor tissue	E vs. Q	600					
HEK	PA vs. no PA	600	+				+
HEK	PA vs. no PA	600	+				+
NGP	PA vs. no PA	600	+				+
NGP	PA vs. no PA	600	+				+
SKNAS	PA vs. no PA	600	+				+
SKNAS	PA vs. no PA	600	+				+
MCF7	PA vs. no PA	600	+				+
MCF7	PA vs. no PA	600	+				+
HEK	PA vs. no PA	50	+		+		+
HEK	PA vs. no PA	50	+		+		+
NGP	PA vs. no PA	50	+		+		+
NGP	PA vs. no PA	50	+		+		+
SKNAS	PA vs. no PA	50	+		+		+
SKNAS	PA vs. no PA	50	+		+		+
MCF7	PA vs. no PA	50	+		+		+
MCF7	PA vs. no PA	50	+		+		+

WT: whole transcriptome; GSP: gene-specific priming; E: Epicentre; Q: Qiagen; RT: reverse transcription.

HEK = HEK-293T FFPE cell pellet RNA; MS2 = MS2 phage carrier RNA.
